# Identification of persistent and resolving subphenotypes of acute hypoxemic respiratory failure in two independent cohorts

**DOI:** 10.1186/s13054-021-03755-7

**Published:** 2021-09-15

**Authors:** Neha A. Sathe, Leila R. Zelnick, Carmen Mikacenic, Eric D. Morrell, Pavan K. Bhatraju, J. Brennan McNeil, Susanna Kosamo, Catherine L. Hough, W. Conrad Liles, Lorraine B. Ware, Mark M. Wurfel

**Affiliations:** 1 Division of Pulmonary, Critical Care and Sleep Medicine, Department of Medicine, University of Washington, 325 9th Avenue, Box # 359640, Seattle, WA 98104 USA; 2 Division of Nephrology, Department of Medicine, University of Washington, Seattle, WA USA; 3 Benaroya Research Institute, Seattle, WA USA; 4 Sepsis Center of Research Excellence, University of Washington, Seattle, WA USA; 5 Division of Allergy, Pulmonary, and Critical Care Medicine, Department of Medicine, Vanderbilt University School of Medicine, Nashville, TN USA; 6 Department of Biochemistry and Molecular Medicine, University of Oulu, Oulu, Finland; 7 Division of Pulmonary and Critical Care, Department of Medicine, Oregon Health and Science University, Portland, OR USA; 8 Division of Allergy and Infectious Diseases, Department of Medicine, University of Washington, Seattle, WA USA; 9 Department of Pathology, Microbiology and Immunology, Vanderbilt University School of Medicine, Nashville, TN USA

**Keywords:** Acute hypoxemic respiratory failure, Mechanical ventilation, Acute lung injury, ARDS, Endophenotypes

## Abstract

**Background:**

Acute hypoxemic respiratory failure (HRF) is associated with high morbidity and mortality, but its heterogeneity challenges the identification of effective therapies. Defining subphenotypes with distinct prognoses or biologic features can improve therapeutic trials, but prior work has focused on ARDS, which excludes many acute HRF patients. We aimed to characterize persistent and resolving subphenotypes in the broader HRF population.

**Methods:**

In this secondary analysis of 2 independent prospective ICU cohorts, we included adults with acute HRF, defined by invasive mechanical ventilation and PaO_2_-to-FIO_2_ ratio ≤ 300 on cohort enrollment (*n* = 768 in the discovery cohort and *n* = 1715 in the validation cohort). We classified patients as *persistent* HRF if still requiring mechanical ventilation with PaO_2_-to-FIO_2_ ratio ≤ 300 on day 3 following ICU admission, or *resolving* HRF if otherwise. We estimated relative risk of 28-day hospital mortality associated with persistent HRF, compared to resolving HRF, using generalized linear models. We also estimated fold difference in circulating biomarkers of inflammation and endothelial activation on cohort enrollment among persistent HRF compared to resolving HRF. Finally, we stratified our analyses by ARDS to understand whether this was driving differences between persistent and resolving HRF.

**Results:**

Over 50% developed persistent HRF in both the discovery (*n* = 386) and validation (*n* = 1032) cohorts. Persistent HRF was associated with higher risk of death relative to resolving HRF in both the discovery (1.68-fold, 95% CI 1.11, 2.54) and validation cohorts (1.93-fold, 95% CI 1.50, 2.47), after adjustment for age, sex, chronic respiratory illness, and acute illness severity on enrollment (APACHE-III in discovery, APACHE-II in validation). Patients with persistent HRF displayed higher biomarkers of inflammation (interleukin-6, interleukin-8) and endothelial dysfunction (angiopoietin-2) than resolving HRF after adjustment. Only half of persistent HRF patients had ARDS, yet exhibited higher mortality and biomarkers than resolving HRF regardless of whether they qualified for ARDS.

**Conclusion:**

Patients with persistent HRF are common and have higher mortality and elevated circulating markers of lung injury compared to resolving HRF, and yet only a subset are captured by ARDS definitions. Persistent HRF may represent a clinically important, inclusive target for future therapeutic trials in HRF.

**Supplementary Information:**

The online version contains supplementary material available at 10.1186/s13054-021-03755-7.

## Background

Acute hypoxemic respiratory failure (HRF) is associated with extended hospital length of stay, functional disability, and increased mortality, but care remains supportive [1–5]. One challenge in identifying effective therapeutics for a broadly defined syndrome like acute HRF is heterogeneity in treatment response and prognosis [6]. Defining reliable subsets of patients with high likelihood of disease-related events or differential treatment responses (often termed subphenotypes) can help target clinical care and trial enrollment to patients most likely to benefit [7, 8]. Previous efforts to identify subphenotypes of respiratory failure have largely centered on acute respiratory distress syndrome (ARDS), which represents less than a quarter of patients on mechanical ventilation, and two-thirds of patients with acute HRF [9–12]. Reliably diagnosing ARDS is also challenging, largely driven by variability in the interpretation and sensitivity of the chest radiograph [13–17]. As such, characterizing subphenotypes of acute HRF is a newly identified research priority by the National Heart, Lung, and Blood Institute with potential to expand inclusion criteria for trials [8,18].

One area of heterogeneity in HRF is the early clinical trajectory. The majority of patients with respiratory failure requiring mechanical ventilation are weaned off within three days [19], while others may develop worsening lung injury and/or excessive fibroproliferation contributing to prolonged need for mechanical ventilation and hypoxemia [20]. Using trajectories of clinical and biologic data to classify subphenotypes has helped identify patients at high risk for poor outcomes in such heterogeneous conditions as chronic obstructive pulmonary disease, pneumonia, and acute kidney injury, and can help determine early biologic events that contribute to subsequent clinical course [10, 21–26].

Our overall goal was to define and characterize a novel subphenotype of acute HRF using persistence of respiratory failure at day 3 following the initiation of mechanical ventilation (*persistent HRF*). The primary objective of this study was to estimate mortality of patients with persistent HRF compared to patients whose respiratory failure and need for mechanical ventilation resolves (*resolving HRF*). The secondary objective was to compare circulating biomarkers previously linked to severity and outcomes in lung injury and profile the early biologic differences between patients with persistent and resolving HRF. In exploratory analyses, we examined relationships between persistent/resolving HRF and ARDS. In particular, we were interested in characterizing the clinical and biologic features of patients with acute HRF who do not fulfill ARDS criteria,  a population that is relatively understudied [27].

## Methods

### Discovery and validation cohorts

We performed a secondary analysis of two prospective observational cohort studies of adults admitted to the intensive care unit (ICU). The discovery cohort was enrolled from medical and surgical ICUs at Harborview Medical Center (Seattle, WA) between 2006 and 2010. Patients were enrolled within 24 hours of ICU admission if meeting criteria for the systemic inflammatory response syndrome [28–30]. Exclusion criteria included admission for trauma; admission for intracranial hemorrhage; severe immunosuppression; and active cancer diagnosis. The validation cohort was enrolled from the medical, surgical, or trauma ICUs at Vanderbilt University Medical Center (Nashville, TN) on the day following admission, from 2006 to 2020, as part of the Validating Acute Lung Injury Biomarkers for Diagnosis (VALID) study [31]. Exclusion criteria included severe chronic lung disease on home supplemental oxygen; cardiac arrest prior to admission; and anticipated discharge from ICU on the day of enrollment. The studies were approved by IRBs at University of Washington and Vanderbilt University. (Additional file 1 has additional cohort description.)

### Study definitions

We restricted analyses to patients with acute HRF, defined as new invasive mechanical ventilation and PaO_2_-to-FIO_2_ ratio (PaO_2_:FIO_2_) ≤ 300 at study enrollment. If PaO_2_:FIO_2_ was unavailable, we used SpO_2_-to-FIO_2_ ratio ≤ 315 [32]. (Additional file 1 has additional details.)

Patients with acute HRF were further classified as persistent or resolving. Persistent HRF was defined by ongoing need for invasive mechanical ventilation and PaO_2_:FIO_2_ ≤ 300 on day 3 following ICU admission. Patients with transient improvements before day 3 (e.g., patients extubated but reintubated, or PaO_2_:FIO_2_ > 300 on day 2) were still classified as persistent HRF as long as they met criteria on day 3. Patients who were extubated or who had PaO_2_:FIO_2_ > 300 on day 3 were classified as resolving HRF. All PaO_2_ measurements were obtained as part of clinical care, and we used the lowest value of PaO_2_:FIO_2_ for each ICU day. To understand how classifying persistent HRF at different early time points altered our results, we performed sensitivity analyses redefining persistent HRF on day 2 after admission (which may fall within the eligibility period of prior trials in respiratory failure) and day 4 (which we hypothesized may better delineate differences in underlying biology) [33–35]. Patients who died before qualifying for persistent HRF were excluded in primary analyses but included in sensitivity analyses. They were classified as persistent HRF if they met the specified criteria on the day of death.

ARDS was defined by the Berlin criteria [36]. Each chest radiograph was reviewed by two Critical Care physicians who came to a consensus on the presence of bilateral opacities consistent with ARDS. The primary outcome was cumulative hospital mortality 28 days following enrollment.

### Biomarker measurements

In both cohorts, plasma samples were collected at enrollment for measurement of biomarkers associated with the development of acute lung injury and ARDS [28, 31]. In the discovery cohort, we measured markers of inflammation and apoptosis (interleukin-6 [IL-6], interleukin-8 [IL-8], soluble tumor necrosis factor receptor-1 [sTNFR-1], soluble Fas [sFas], interleukin-17A [IL-17A], granulocyte colony-stimulating factor [G-CSF]) as well as markers of endothelial dysfunction/activation (angiopoietin-2 [Ang-2], angiopoietin-1 [Ang-1], and soluble vascular cell adhesion protein-1 [sVCAM-1]) using a electrochemiluminiscent immunoassay (MesoScale Discovery, Rockville, MD). Of these markers, IL-6, IL-8, sTNFR-1, and Ang-2 were also measured in the validation cohort. IL-6 and IL-8 were measured on the same platform, while sTNFR-1 and Ang-2 were measured by ELISA (R&D Systems, Minneapolis, MN).

### Statistical analyses

We reported demographics, baseline comorbidities, ICU conditions, and outcomes by persistent and resolving HRF. Continuous variables were reported as medians with interquartile ranges and compared using Mann–Whitney U tests, while categorical variables were reported as counts with percentage and compared using Chi-squared tests.

Our primary analysis estimated relative risk (RR) of mortality associated with persistent HRF compared to resolving HRF using generalized linear models with Poisson distribution and robust standard errors [37]. In adjusted models, we included age, sex, chronic respiratory disease, and either baseline PaO_2_:FIO_2_ or modified acute physiology and chronic health evaluation (APACHE) score as prespecified confounders. We used APACHE-III in the discovery cohort and APACHE-II in the validation cohort [38, 39]. We did not include both PaO_2_:FIO_2_ and APACHE score in the same model to avoid multicollinearity. To further select for a population of comparable HRF severity at baseline, we performed analyses restricting to patients with PaO_2_:FIO_2_ < 150 and another excluding patients with chronic lung disease.

In our secondary analysis, we used linear regression with adjustments as above to estimate the fold difference in geometric mean concentrations of each biomarker between persistent and resolving HRF. We used geometric means and log_2_-transformed biomarker measurements due to right skew.

We performed two exploratory analyses. First, we examined whether ARDS, identified at any point by ICU day 3, was driving differences between persistent and resolving HRF. We stratified persistent/resolving HRF by ARDS (± ARDS) to create 4 groups: (1) resolving HRF/-ARDS, (2) resolving HRF/+ARDS, (3) persistent HRF/-ARDS, and (4) persistent HRF/+ARDS. We compared RR of mortality in each group to the reference of resolving HRF/−ARDS using the methods from our primary analysis. In a second exploratory analysis, we examined whether previously described hyperinflammatory and hypoinflammatory subphenotypes have any relationship to persistent HRF [9, 40–43]. These subphenotypes have been independently validated in ARDS and HRF, and were associated with distinct prognoses. We classified patients as hypoinflammatory or hyperinflammatory using a logistic regression model of plasma sTNFR-1, IL-8, and bicarbonate as previously reported [44], and then compared the proportion of patients who developed persistent HRF in each group.

Observations with missing covariates were excluded from each model. Analyses were conducted in STATA (version 16.0).

## Results

### Cohort description

We identified 768 patients with acute HRF in the discovery cohort, and 1715 patients in the validation cohort (Additional file 1: Figures S1–S2). In the discovery cohort, 386 patients (50%) developed persistent HRF; in the validation cohort, 1032 (60%) patients developed persistent HRF. Baseline characteristics and outcomes by persistent and resolving HRF are summarized in Table 1. Patients with persistent HRF had higher illness severity and a higher proportion of ARDS on enrollment compared to patients with resolving HRF. Patients with persistent HRF also had fewer VFD and longer hospital lengths of stay, with the median differences exceeding what was expected based on how persistent HRF was defined. We had limited data regarding initial ventilator management (Additional file 1: Table S1), but noted patients with persistent HRF were treated with modestly higher FIO_2_ than patients with resolving HRF in the discovery cohort, and all patients in both cohorts were on at least 5 cm H_2_O positive end-expiratory pressure.

**Table 1:** Cohort descriptions by persistent and resolving hypoxemic respiratory failure

	Discovery cohort			Validation cohort		
	Resolving *N* = 382	Persistent *N* = 386	*P* value	Resolving *N* = 683	Persistent *N* = 1032	*P* value
*Demographics*						
Age, years	54 (45–63)	53 (44–64)	0.780	55 (41–66)	53 (41–64)	0.131
Female sex	144 (38%)	129 (33%)	0.216	264 (39%)	357 (35%)	0.087
Race			0.013			0.004
White	271 (76%)	291 (80%)		573 (84%)	910 (88%)	
*Black/African American*	50 (14%)	26 (7%)		99 (14%)	98 (10%)	
*Other*	61 (16%)	69 (18%)		11 (2%)	24 (2%)	
*Baseline comorbidities*						
Diabetes	113 (30%)	112 (29%)	0.863	186 (27%)	258 (25%)	0.301
Cirrhosis	44 (12%)	44 (11%)	0.959	61 (9%)	74 (7%)	0.185
Chronic respiratory disease	60 (16%)	88 (23%)	0.013	134 (20%)	122 (12%)	< 0.001
Heart Failure	41 (11%)	43 (11%)	0.857	74 (11%)	83 (8%)	0.050
Alcohol Use Disorder	115 (30%)	135 (35%)	0.150	139 (20%)	240 (23%)	0.156
*ICU events on enrollment*						
Type of ICU			0.423			< 0.001
*Medical*	260 (68%)	273 (71%)		305 (45%)	409 (40%)	
*Surgical*	122 (32%)	113 (29%)		164 (24%)	192 (19%)	
*Trauma*	0 (0%)	0 (0%)		210 (31%)	429 (42%)	
Shock	87 (23%)	191 (49%)	< 0.001	261 (38%)	532 (52%)	< 0.001
Sepsis	298 (78%)	330 (85%)	0.007	261 (38%)	475 (46%)	0.001
Pneumonia	81 (21%)	143 (37%)	< 0.001	198 (29%)	385 (37%)	< 0.001
Acute respiratory distress syndrome	51 (13%)	124 (32%)	< 0.001	145 (21%)	393 (38%)	< 0.001
*Illness severity on enrollment*						
PaO_2_-to-FIO_2_ ratio	190 (138–254)	128 (85–207)	< 0.001	187 (136–236)	147 (97–205)	< 0.001
SOFA	4 (3–6)	5 (4–7)	< 0.001	8 (7–10)	10 (8–11)	< 0.001
APACHE-III	53 (36–72)	69 (49–90)	< 0.001	n.a	n.a	
APACHE-II	n.a	n.a		26 (21–31)	29 (23–34)	< 0.001
*Outcomes*						
Ventilator free days	25 (23–26)	15 (0–21)	< 0.001	25 (23–26)	17 (0–22)	< 0.001
Hospital length of stay, days	10 (6–19)	19 (12–32)	< 0.001	10 (7–18)	16 (10–26)	< 0.001
Mortality	31 (8%)	77 (20%)	< 0.001	70 (10%)	230 (22%)	< 0.001

*ICU *intensive care unit, *APACHE* acute physiology and chronic health evaluation, *SOFA* sequential organ failure assessment, *n.a.* not applicable. Continuous variables are expressed as median (interquartile range) and categorical variables are expressed as number (percentage). *P* values correspond to Mann–Whitney U tests for continuous variables and Chi-squared tests for categorical variables

### Mortality in persistent/resolving HRF

Mortality was significantly higher among patients with persistent HRF, compared to those with resolving HRF (20% vs. 8% in discovery; 22% vs. 10% in validation). Adjusting for age, sex, chronic respiratory disease, and APACHE-III, persistent HRF carried a 1.68-fold higher (95% CI 1.11, 2.54) risk of death in the discovery cohort (Table 2). In the validation cohort, persistent HRF was associated with a 1.93-fold higher (95% CI 1.50, 2.47) risk of death in a similarly adjusted model. Fewer than 5% of patients died before day 3 in either cohort and were excluded from primary analyses; the findings were similar when we included these patients (Additional file 1: Table S2). The association between persistent HRF and mortality was robust to other sensitivity analyses, including redefining persistent HRF at day 2 and day 4, as well as restricting the population to enrollment PaO_2_:FIO_2_ < 150 and those without chronic lung disease (Additional file 1: Tables S3–S6).

**Table 2:** Relative risk of mortality associated with persistent hypoxemic respiratory failure

	Deaths	Relative risk (95% confidence interval)		
	*N* (%)	Unadjusted	Model A	Model B
*Discovery cohort*				
Resolving	31 (8%)	1.00 (reference)	1.00 (reference)	1.00 (reference)
Persistent	77 (20%)	2.46 (1.66, 3.64)^a^	2.34 (1.56, 3.49)^a^	1.68 (1.11, 2.54)^b^
*Validation cohort*				
Resolving	70 (10%)	1.00 (reference)	1.00 (reference)	1.00 (reference)
Persistent	230 (22%)	2.17 (1.69, 2.79)^a^	2.08 (1.58, 2.76)^a^	1.93 (1.50, 2.47)^a^

Mortality is in-hospital mortality 28 days after enrollment

Model A: adjusted for age, sex, chronic respiratory disease, and PaO_2_-to-FIO_2_ ratio on enrollment

Model B: adjusted for age, sex, chronic respiratory disease, and modified acute physiology and chronic health evaluation on enrollment (APACHE-III score in discovery cohort and APACHE-II score in validation cohort)

^a^*p* < 0.001 ^b^*p* <  0.05

### Circulating biomarkers in persistent/resolving HRF

**Fig. 1:**
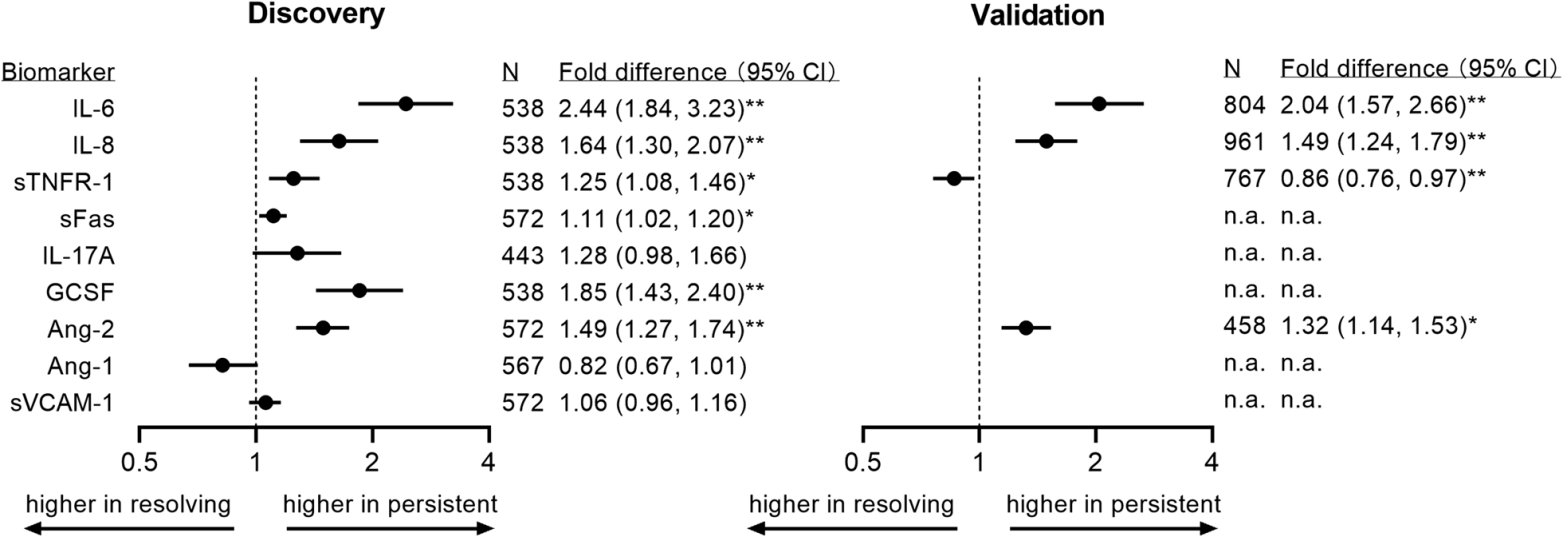
Adjusted differences in biomarker concentrations between persistent and resolving hypoxemic respiratory failure. Abbreviations: IL-6 = interleukin-6; IL-8 = interleukin-8; sTNFR-1 = soluble tumor necrosis factor receptor-1; sFas = soluble Fas; IL-17A = interleukin-17A; G-CSF = granulocyte colony-stimulating factor; Ang-2 = angiopoietin-2; Ang-1 = angiopoietin-1; sVCAM-1 = soluble vascular cell adhesion protein-1; n.a. = not applicable (not measured in specified cohort). N specifies number of patients who had that biomarker measured in each cohort. Fold differences reflect ratio of geometric mean concentrations among patients with persistent hypoxemic respiratory failure, to patients with resolving hypoxemic respiratory failure. Fold differences are adjusted for age, sex, chronic respiratory disease, and acute physiology and chronic health evaluation (APACHE-III in discovery cohort and APACHE-II in validation cohort). ** *P* < 0.001 * *P* < 0.05

We next compared enrollment biomarker profiles to see whether persistent and resolving HRF exhibited distinctive early patterns of inflammation and/or lung injury (Additional file 1: Tables S7–S8). In the discovery cohort, biomarkers of inflammation were higher in patients with persistent HRF compared to resolving HRF, including IL-6 (2.44-fold [95% CI 1.84, 3.23]), G-CSF (1.85-fold [95% CI 1.43, 2.40]), IL-8 (1.64-fold [95% CI 1.30, 2.07]), and sTNFR-1 (1.25-fold [95% CI 1.08, 1.46]) even after adjusting for age, sex, chronic respiratory disease, and APACHE (Fig. 1). A marker of apoptotic pathways, sFas, was 1.11-fold higher (95% CI 1.02, 1.20) in persistent HRF compared to resolving HRF. Ang-2, reflecting endothelial injury, was 1.49-fold higher (95% CI 1.27, 1.74) in persistent HRF.

We replicated the finding that persistent HRF is associated with greater inflammation (higher IL-6, IL-8) and endothelial injury (higher Ang-2) compared to resolving HRF in the validation cohort (Fig. 1). Only sTNFR-1 was found to be 14% lower among persistent HRF compared to resolving HRF (0.86-fold, 95% CI 0.76, 0.97) in APACHE-adjusted models; however, the difference was not significant in other unadjusted or adjusted analyses (Additional file 1: Tables S7–S8).

Sensitivity analyses produced similar results (Additional file 1: Tables S9–S13). Of note, redefining persistent HRF at day 2 attenuated differences in some biomarkers, while redefining persistent HRF at day 4 and restricting analyses to patients with PaO_2_:FIO_2_ < 150 magnified biologic differences between groups.

### Persistent/resolving HRF stratified by ARDS

Additional file 1: Table S14 lists features of patients with persistent and resolving HRF, stratified by whether or not they met ARDS criteria at any point by day 3. Of note, half of patients with persistent HRF did not meet ARDS criteria.

In the discovery cohort, mortality was highest among persistent HRF/+ARDS (25%), followed by persistent HRF/-ARDS (15%), resolving HRF/+ARDS (10%), and resolving HRF/-ARDS (7%) (Table 3). Persistent HRF both with and without ARDS were associated with a higher risk for death compared to resolving HRF/-ARDS, although adjustment for APACHE-III attenuated estimates. Resolving HRF/+ARDS did not have a significantly higher risk for death compared to resolving HRF/−ARDS. In the validation cohort, all 3 comparator groups had over twofold higher risk for death than resolving HRF/−ARDS.

**Table 3:** Relative risk of mortality associated with persistent and resolving hypoxemic respiratory failure, stratified by acute respiratory distress syndrome (± ARDS)

		Total	Deaths	Relative risk (95% confidence interval)		
		*N*	*N* (%)	Unadjusted	Model A	Model B
*Discovery cohort*						
Resolving	−ARDS	296	22 (7%)	1.00 (reference)	1.00 (reference)	1.00 (reference)
	+ARDS	86	9 (10%)	1.41 (0.67, 2.94)	1.36 (0.65, 2.83)	0.96 (0.49, 1.89)
Persistent	−ARDS	186	28 (15%)	2.03 (1.19, 3.43)^b^	2.03 (1.20, 3.43)^b^	1.48 (0.86, 2.52)
	+ARDS	200	49 (25%)	3.30 (2.06, 5.28)^a^	3.09 (1.91, 5.02)^a^	1.81 (1.07, 3.08)^c^
*Validation cohort*						
Resolving	−ARDS	515	37 (7%)	1.00 (reference)	1.00 (reference)	1.00 (reference)
	+ARDS	168	33 (20%)	2.73 (1.77, 4.23)^a^	2.48 (1.53, 4.02)^a^	2.50 (1.65, 3.80)^a^
Persistent	−ARDS	559	114 (20%)	2.84 (2.00, 4.03)^a^	2.59 (1.77, 3.79)^a^	2.52 (1.78, 3.58)^a^
	+sARDS	473	116 (25%)	3.41 (2.41, 4.84)^a^	3.19 (2.17, 4.69)^a^	2.93 (2.07, 4.16)^a^

Mortality is in-hospital mortality 28 days after enrollment

+ARDS refers to patients who were adjudicated as ARDS at any point by ICU day 3; −ARDS are patients who were not adjudicated to have ARDS by ICU day 3

Model A: adjusted for age, sex, chronic respiratory disease, and PaO_2_-to-FIO_2_ ratio on enrollment

Model B: adjusted for age, sex, chronic respiratory disease, and modified acute physiology and chronic health evaluation on enrollment (APACHE-III score in discovery cohort and APACHE-II score in validation cohort)

^a^*p* < 0.001 ^b^*p* < 0.01 ^c^*p* < 0.05

**Fig. 2:**
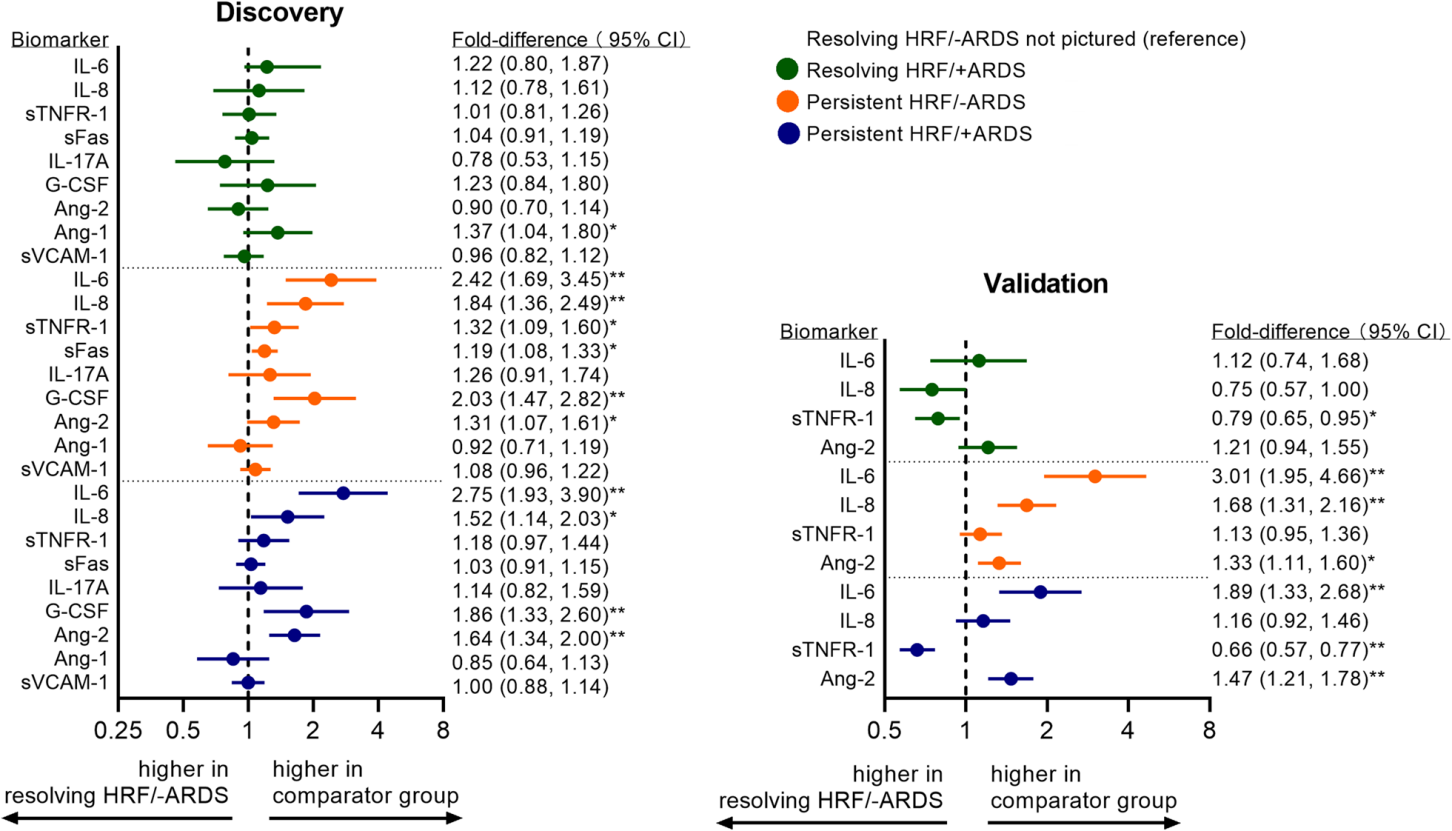
Adjusted differences in biomarker concentrations between persistent and resolving hypoxemic respiratory failure, stratified by acute respiratory distress syndrome (± ARDS). Abbreviations: IL-6 = interleukin-6; IL-8 = interleukin-8; sTNFR-1 = soluble tumor necrosis factor receptor-1; sFas = soluble Fas; IL-17A = interleukin-17A; G-CSF = granulocyte colony-stimulating factor; Ang-2 = angiopoietin-2; Ang-1 = angiopoietin-1; sVCAM-1 = soluble vascular cell adhesion protein-1; n.a. = not applicable (not measured in specified cohort); ARDS = acute respiratory distress syndrome; HRF = hypoxemic respiratory failure. Fold differences reflect ratio of geometric mean concentrations among patients in each specified group, to patients with resolving hypoxemic respiratory failure without ARDS (resolving HRF/-ARDS). Fold differences are adjusted for age, sex, chronic respiratory disease, and modified acute physiology and chronic health evaluation (APACHE-III in discovery cohort and APACHE-II in validation cohort). ** *P* < 0.001 * *P* < 0.05

Patients with persistent HRF, both with and without ARDS, exhibited similar biomarker profiles characterized by inflammation and endothelial injury (Additional file 1: Table S15). IL-6, IL-8, and Ang-2 concentrations were significantly higher among persistent HRF/+ARDS and persistent HRF/-ARDS in both discovery and validation cohorts, compared to resolving HRF/-ARDS (Fig. 2). In contrast, the biomarker profile among patients with resolving HRF/+ARDS was not significantly different from resolving HRF/-ARDS. sTNFR-1 was lower among both +ARDS strata compared to resolving HRF/-ARDS in the validation cohort, but this difference was not seen in discovery.

Furthermore, we were interested in comparing resolving HRF/+ARDS to persistent HRF/-ARDS, expecting some elevations in lung injury biomarkers among +ARDS patients. We performed pairwise comparisons across all 4 strata with Bonferroni correction (Additional file 1: Table S16). We found persistent HRF/-ARDS patients consistently had higher markers of inflammation and endothelial dysfunction, while resolving HRF/+ARDS had higher Ang-1, a marker of endothelial stabilization.

### Persistent/resolving HRF and hyper/hypoinflammatory subphenotypes

We classified the subset of patients with bicarbonate, IL-8, and sTNFR-1 measured as hypoinflammatory or hyperinflammatory (Additional file 1: Figure S3). The distribution and features of patients in each were consistent with prior descriptions of these subphenotypes in both ARDS and non-ARDS respiratory failure [9, 40–43], with mortality among hyperinflammatory patients higher than hypoinflammatory patients (Additional file 1: Table S17).

Patients with hyperinflammatory HRF were more likely to develop persistent HRF compared to patients with hypoinflammatory HRF in the discovery (61% vs. 45%, *P* = 0.001) and validation cohorts (79% vs. 63% *P* < 0.001). However, since few patients were classified as hyperinflammatory, far more hypoinflammatory patients than hyperinflammatory developed persistent HRF.

## Discussion

In two independent cohorts of acute HRF, we found that patients with persistent HRF 3 days following ICU admission had a markedly higher risk for subsequent in-hospital mortality compared to patients with resolving HRF, even after adjustment for initial illness severity. Cumulative incidence of persistent HRF was over 50% in both cohorts, which included a large, diverse population of patients from medical, surgical, and trauma ICUs. Patients with persistent HRF had evidence of greater systemic inflammation and endothelial dysfunction/activation on enrollment compared to resolving HRF. Finally, we saw that these findings were not driven only by patients with ARDS; rather, patients with persistent HRF who did not meet ARDS criteria represented a prevalent group with poor prognosis and elevated markers of lung injury. Overall, this work illustrates that ARDS is not merely one end of the spectrum of disease severity in acute HRF and that characterizing clinical and biologic features associated with persistent HRF regardless of ARDS diagnosis may generate new insight into what drives outcomes in respiratory failure.

We observed significant differences in mortality, VFD, and length of stay between persistent and resolving HRF. While the findings may in part reflect the milder illness among patients with resolving HRF at enrollment, we hypothesize patients with persistent and resolving HRF have other underlying reasons to explain differing trajectories. Supporting this hypothesis, the risk of death was different even after adjusting for baseline PaO_2_:FIO_2_ and APACHE score. Furthermore, our sensitivity analysis of patients with PaO_2_:FIO_2_ < 150 diminished differences in initial illness severity while strengthening the effect of persistent HRF on subsequent mortality. In fact, over half of the resolving HRF patients in our study had PaO_2_:FIO_2_ < 150 at baseline, indicating that many patients who begin their ICU course with serious respiratory failure improve quickly. The prognostic value of this early trajectory, and the disconnect between initial severity of respiratory failure and subsequent outcomes, has not previously been investigated in acute HRF but has been described in ARDS [10, 21, 45–47]. Notably, a secondary analysis of randomized trial data showed patients with ARDS that rapidly improved within 1 day had substantially lower mortality than patients with persistent ARDS and that 63% of rapidly improving ARDS patients were moderate or severe at enrollment [10]. A study from the international multicenter LUNG SAFE cohort reported that 82% of patients with mild ARDS at enrollment have persistent or worsening ARDS in the first week of onset and that patients with persistent/worsening ARDS on ICU day 2 have higher hospital mortality compared to those with resolving ARDS [46]. In general, the PaO_2_:FIO_2_ at onset of respiratory failure is not as consistently associated with overall mortality as PaO_2_:FIO_2_ measured at later time points, and in our study too, we saw the overall mortality among patients with enrollment PaO_2_:FIO_2_ < 150 was similar to that of the entire cohort [48]. These data all support an approach that incorporates reassessing respiratory failure on ICU day 3 to define a subphenotype that may be more patho-physiologically homogenous and at high risk for poor outcomes. Further work is needed to understand which underlying factors contribute to the development of persistent or resolving HRF and their associated differences in mortality, which may relate to treatment (e.g., fluid management, lung-protective ventilation), the patient (e.g., genetic susceptibility to lung injury, chronic comorbidity), and/or the acute illness itself (e.g., pathogen, type of trauma).

Enrollment of patients with persistent HRF into clinical trials of therapies for respiratory failure could help select for patients at higher risk for disease-related outcomes, a strategy known as prognostic enrichment [7]. There is motivation to identify new subsets for prognostic enrichment, since studies of ARDS populations suggest mortality attributable to respiratory failure alone is relatively low [49, 50]. Similarly, identifying patients with resolving HRF may help avoid exposing them to potentially unnecessary, costly, or toxic new therapies. One strength of our approach is that persistent and resolving HRF can be classified with commonly obtained clinical data, unlike other enrichment strategies requiring measurements only available in research settings [9, 11]. In addition, we showed that persistent HRF selects for a high-risk population as early as 48 hours after mechanical ventilation, falling into the enrollment window of recent trials and accommodating early therapies for respiratory failure [51].

Another important strategy to improve therapeutic trials is selecting patients with shared biologic mechanisms that are likely to respond to a specific therapy, known as predictive enrichment. Although we do not directly examine whether persistent and resolving HRF have differential responses to therapy, we do show that patients with persistent HRF have higher systemic inflammation and endothelial dysfunction. These patients could be targeted for such emerging therapeutic strategies as early immune modulation and endothelial stabilization/repair [52]. Additionally, persistent HRF enriches for the hyperinflammatory subphenotype of respiratory failure, which among ARDS patients has been associated with differential response to fluid management, statin therapy, and ventilator strategy [40, 41, 53].

Most notably, we showed that 48% of persistent HRF patients in the discovery cohort and 54% in the validation cohort have high mortality and elevated circulating markers of lung injury, yet are not captured in ARDS definitions. Data on these patients with acute HRF who do not meet ARDS criteria remain limited. One large epidemiologic study of acute respiratory failure from Northern Europe also showed patients who did and did not meet ARDS criteria had similar mortality, although this study was done before the dissemination of lung-protective ventilation and the development of the Berlin definition [5]. Newer work from the LUNG SAFE cohort examined acute HRF patients with new pulmonary infiltrates and also found that patients with unilateral infiltrates (i.e., without ARDS) had similar adjusted mortality to patients with ARDS [27]. In our study, we suspect that the main factor distinguishing ± ARDS was the chest radiograph, which has been a source of misclassification, measurement burden, and interobserver variability in ARDS diagnosis [13–16, 54, 55]. Our paper builds upon this existing work by also showing that patients with persistent HRF without ARDS had a biomarker profile more consistent with lung injury than ARDS patients with resolving HRF. Non-ARDS patients with persistent HRF may share biologic features with ARDS and thereby may benefit from therapies under investigation for ARDS.

This study has several limitations. First, while we used a broad range of baseline clinical information to control for differences between groups, there may be residual confounding due to unmeasured differences in early clinical care. Second, while the biomarkers measured have been associated with ARDS outcomes and severity, they may not be lung-specific and could reflect processes outside of the lung. Third, the platforms used to measure these biomarkers were not the same in both cohorts. While we would expect this difference to bias our results to the null, it could partially explain discrepancies between cohorts in the relationship between sTNFR-1 and persistent HRF. Fourth, while PaO_2_:FIO_2_ is an established measure of the severity of respiratory failure, it is subject to change with ventilator management independent of changes in clinical status [48, 56], and captures only a portion of physiologic derangements in HRF [56, 57]. Although such differences in ventilator management could have unpredictable effects on classification of persistent HRF, the replication of our main findings in cohorts from two independent medical centers suggests the findings are robust to at least some variability in clinical care. Finally, the identification of persistent HRF is based on a trajectory and cannot be used to classify patients at illness onset. Areas of future research include developing approaches for early discrimination of persistent HRF to aid trial enrollment and designing prospective studies to better understand the mechanisms differentiating persistent from resolving HRF.

## Conclusions

Among mechanically ventilated patients with acute HRF, the subphenotype of persistent HRF on ICU day 3 identifies patients with high mortality and prolonged respiratory failure. Patients with persistent HRF have distinctive features early in the course of illness, such as elevated markers of inflammation and endothelial dysfunction. Patients with persistent HRF, both with and without ARDS, may warrant inclusion in trials of targeted therapeutics for respiratory failure.

## Supplementary Information

**Additional file 1.** Supplemental methods, figures, and tables.

## Data Availability

The datasets used and/or analyzed during the current study are available from the corresponding authors on reasonable request.

## Code availability

The code used during the current study is available from the corresponding author on reasonable request.
