# Supplementary material for: Identification of persistent and resolving subphenotypes of acute hypoxemic respiratory failure in two independent cohorts
Source: Crit Care. 2021 Sep 15;25:336. doi: 10.1186/s13054-021-03755-7 (PMC8442814; doi:10.1186/s13054-021-03755-7)
Supplement: Supplementary file 1 — Additional file 1. Supplemental methods, figures, and tables. [file 13054_2021_3755_MOESM1_ESM.pdf]

Additional File 1: SUPPLEMENTAL METHODS, FIGURES, AND TABLES

Manuscript Title: Identification of persistent and resolving subphenotypes of acute hypoxemic respiratory failure in two independent cohorts

Authors: Neha A. Sathe, MD*[1]; Leila R. Zelnick, PhD[2]; Carmen Mikacenic, MD[1,3]; Eric D. Morrell, MD, MA[1]; Pavan K. Bhatraju, MD, MSc[1,4]; J. Brennan McNeil, MS[5]; Susanna Kosamo, PhD[6]; Catherine L. Hough, MD, MSc[7]; W. Conrad Liles, MD, PhD[4,8]; Lorraine B, Ware, MD[5,9]; Mark M. Wurfel, MD, PhD[1,4].

Affiliations: [1]Division of Pulmonary, Critical Care and Sleep Medicine, Department of Medicine, University of Washington, Seattle, WA; [2]Division of Nephrology, Department of Medicine, University of Washington, Seattle, WA; [3]Benaroya Research Institute, Seattle, WA; [4]Sepsis Center of Research Excellence, University of Washington; [5]Division of Allergy, Pulmonary, and Critical Care Medicine, Department of Medicine, Vanderbilt University School of Medicine, Nashville, TN, USA; [6]Department of Biochemistry and Molecular Medicine, University of Oulu, Finland; [7]Division of Pulmonary and Critical Care, Department of Medicine, Oregon Health & Science University, Portland, OR, USA; [8]Division of Allergy and Infectious Diseases, Department of Medicine, University of Washington, USA, [9]Department of Pathology, Microbiology and Immunology, Vanderbilt University School of Medicine, Nashville, TN.

***To whom correspondence should be addressed:**
Neha A. Sathe, MD
325 9th Avenue
Seattle, WA 98104
Telephone #: 206-744-3238. Email: nas212@uw.edu

SUPPLEMENTAL METHODS

Discovery and Validation Cohort Description

Patients were enrolled from the medical and surgical intensive care units (ICU) at Harborview Medical Center (Seattle, WA) between 2006 and 2010 in the discovery cohort, known as HMC-SIRS. Patients were enrolled within 24 hours of ICU admission if they met criteria for the systemic inflammatory response syndrome(1). Exclusion criteria were admission for trauma; admission for intracranial hemorrhage; severe immunosuppression; and active cancer diagnosis.

The validation cohort, called the Validating Acute Lung Injury Biomarkers for Diagnosis (VALID) cohort, included patients enrolled from January 2006 to December 2020 at Vanderbilt University Medical Center. For this analysis we included patients admitted to either the medical, surgical, or trauma ICUs, and excluded patients admitted to the cardiothoracic ICU. Patients were enrolled into VALID on the day following ICU admission. Exclusion criteria for the VALID cohort included severe chronic lung disease (e.g. severe asthma, severe chronic obstructive pulmonary disease, or pulmonary fibrosis); admission for uncomplicated drug overdose; admission to another ICU > 3 days prior to enrollment; cardiac arrest prior to admission; and anticipated discharge from ICU on the day following admission.

Assessment of hypoxemic respiratory failure

As described in the main text, we primarily used a PaO2 to FIO2 ratio (PaO2:FIO2) ≤ 300 to classify patients as hypoxemic. In the discovery cohort, of 890 patients initiated on mechanical ventilation on enrollment, 11 patients (1.2%) did not have PaO2:FiO2. No clear SpO2 to FIO2 ratios (SpO2:FIO2) were available, so we used SpO2 < 92% while on invasive mechanical ventilation to classify patients as hypoxemic. In the validation cohort, of 2339 patients who were mechanically ventilated on enrollment, 380 (16%) did not have PaO2:FIO2 available. Consistent with approaches used in other studies of acute respiratory distress syndrome, we used SpO2:FIO2 ≤ 315 to determine hypoxemia (2).

REFERENCES

1. Singer M, Deutschman CS, Seymour CW, Shankar-Hari M, Annane D, Bauer M, et al. The Third International Consensus Definitions for Sepsis and Septic Shock (Sepsis-3). JAMA. 2016 Feb 23;315(8):801–10.

2. Rice TW, Wheeler AP, Bernard GR, Hayden DL, Schoenfeld DA, Ware LB. Comparison of the Spo2/Fio2 Ratio and the Pao2/Fio2 Ratio in Patients With Acute Lung Injury or ARDS. Chest. 2007 Aug 1;132(2):410–7.

SUPPLEMENTAL FIGURES

Figure S1: Study flow diagram for discovery cohort

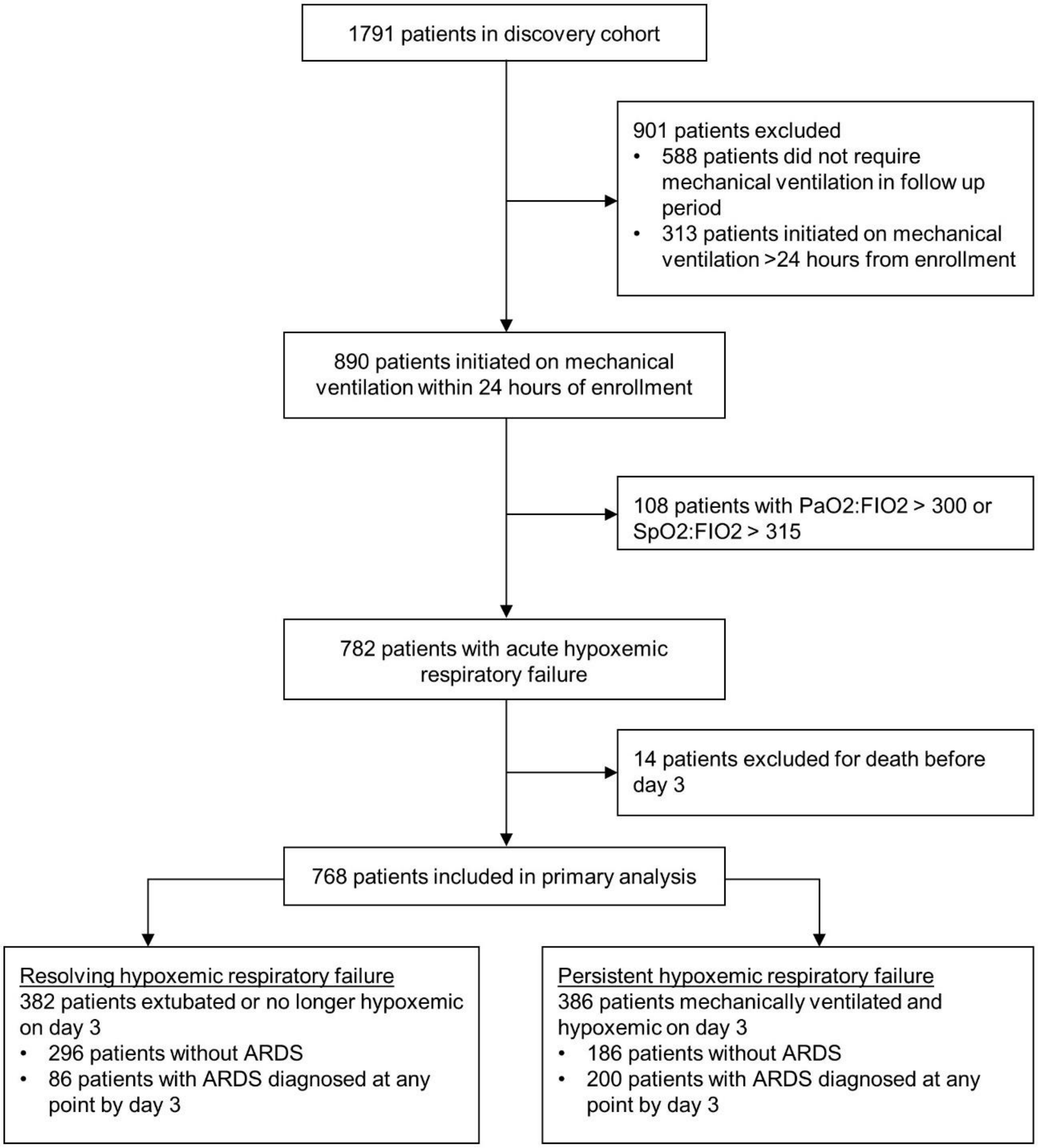

Figure S2: Study flow diagram for validation cohort

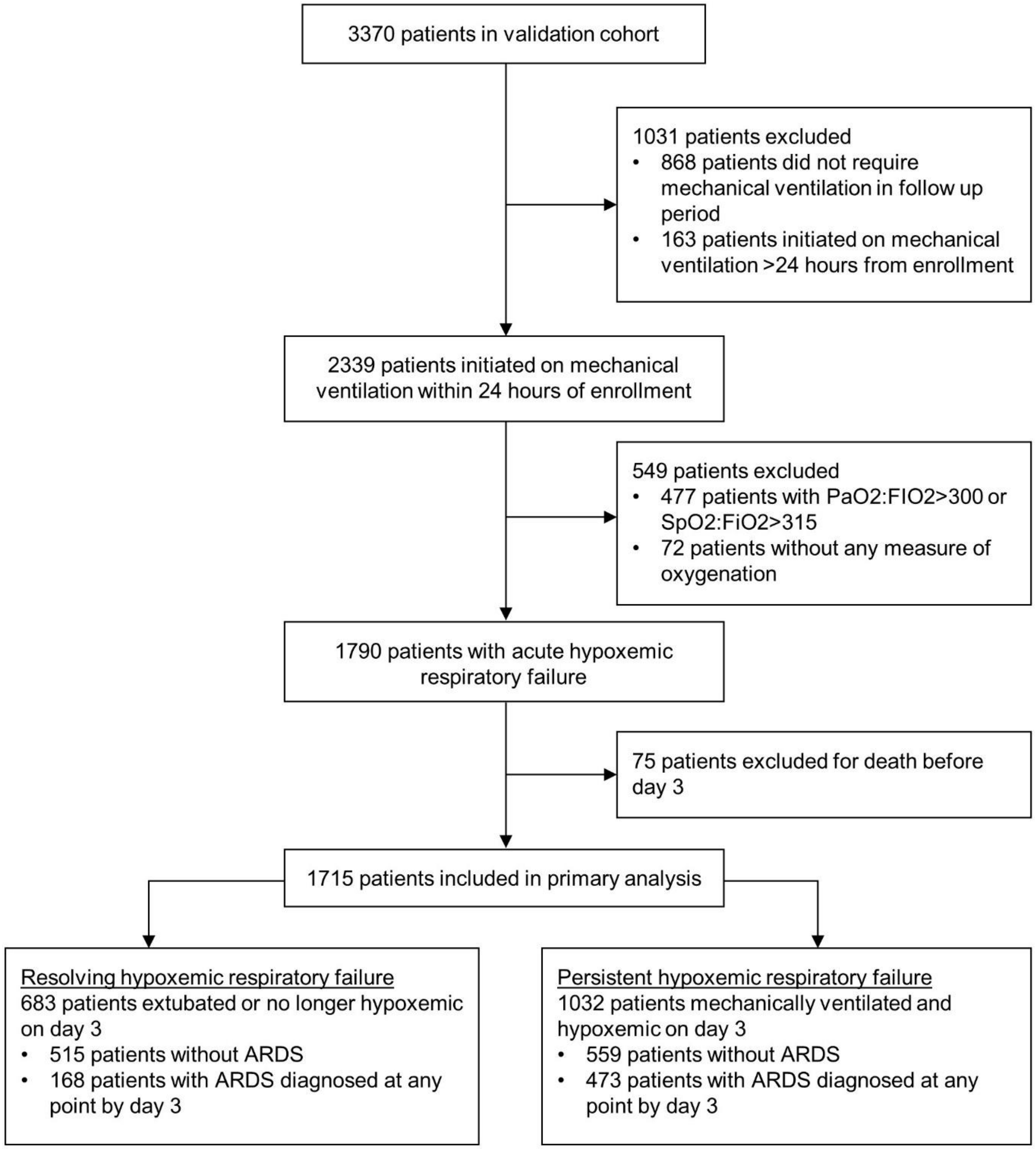

Figure S3. Sankey diagram from hypoinflammatory/hyperinflammatory subphenotypes at enrollment to persistent/resolving hypoxemic respiratory failure at day 3

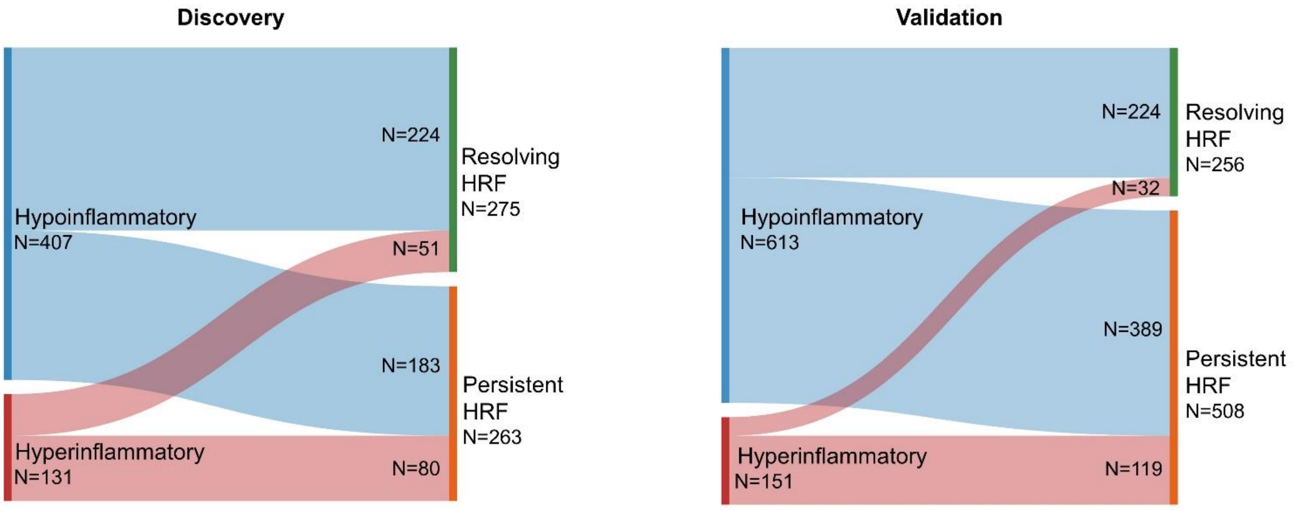

Abbreviations. HRF = hypoxemic respiratory failure.

Hyperinflammatory and hypoinflammatory subphenotypes were classified using a three-variable model of bicarbonate, interleukin-8, and soluble tumor necrosis factor receptor-1 in 538 patients in the discovery cohort and 764 patients in the validation cohort. Hyperinflammatory patients were more likely to develop persistent HRF compared to hypoinflammatory patients in the discovery (80/131, 61% vs. 183/407, 45%, $P = 0.001$) and validation cohorts (119/151, 79% vs. 389/613, 63% $P < 0.001$) by Chi square testing. However, the absolute number of hypoinflammatory patients who developed persistent HRF was high in both cohorts. The hypoinflammatory patients who developed persistent HRF also had moderately high mortality (80/183, 16% in discovery; 86/389, 22% in validation), although not as high as hyperinflammatory patients who developed persistent HRF (22/80, 28% in discovery; 40/119, 34% in validation).

SUPPLEMENTAL TABLES – VENTILATOR DATA

Table S1: Ventilator data on cohort enrollment by persistent and resolving HRF

	Discovery Cohort			Validation Cohort		
	**Resolving**	**Persistent**	***P* value**	**Resolving**	**Persistent**	***P* value**
FIO_2, %	50 (40-55)	60 (50-80)	< 0.001	n.a.	n.a.	n.a.
PEEP, cm H_2O	5 (5-5)	5 (5-8)	0.017	10 (5-10)	10 (8-12)	<0.001
Tidal volume, mL	556 (500-600)	537 (482-600)	0.154	450 (400-500)	465 (405-500)	0.004

Median (interquartile range) of each parameter is displayed. P values reflect Wilcoxon rank sum tests.
FIO_2 fractional inspired oxygen, data available in all patients in discovery cohort but unavailable in the validation cohort.
PEEP positive end expiratory pressure, data available on 223 patients in discovery cohort and 533 patients in the validation cohort. In data available, no patient was on less than 5 cm H_2O PEEP.
Tidal volume available in 219 patients in discovery cohort and 1686 patients in the validation cohort.

SUPPLEMENTAL TABLES – SENSITIVITY ANALYSES OF MORTALITY

Table S2: Relative risk of mortality associated with persistent hypoxemic respiratory failure, inclusive of patients who died before day 3

	Total	Deaths	Relative risk (95% Confidence Interval)		
	N	*N (%)*	*Unadjusted*	*Model A*	*Model B*
**Discovery Cohort**					
Resolving	383	32 (8%)	1.00 (reference)	1.00 (reference)	1.00 (reference)
Persistent	399	90 (23%)	2.70 (1.85, 3.94)	2.52 (1.71, 3.72)	1.94 (1.31, 2.87)
**Validation Cohort**					
Resolving	685	72 (11%)	1.00 (reference)	1.00 (reference)	1.00 (reference)
Persistent	1105	303 (27%)	2.61 (2.05, 3.31)	2.41 (1.84, 3.14)	2.20 (1.74, 2.79)

Mortality is in-hospital mortality 28 days after enrollment.
Model A: adjusted for age, sex, chronic respiratory disease, and PaO2 to FIO2 ratio on enrollment.
Model B: adjusted for age, sex, chronic respiratory disease, and modified acute physiology and chronic health evaluation on enrollment (APACHE-III score in discovery cohort and APACHE-II score in validation cohort).

Table S3: Relative risk of mortality associated with persistent hypoxemic respiratory failure, redefined at day 2

	Total	Deaths	Relative risk (95% Confidence Interval)		
	N	*N (%)*	*Unadjusted*	*Model A*	*Model B*
**Discovery Cohort**					
Resolving	333	28 (8%)	1.00 (reference)	1.00 (reference)	1.00 (reference)
Persistent	442	87 (20%)	2.34 (1.57, 3.50)	2.15 (1.41, 3.28)	1.58 (1.04, 2.38)
**Validation Cohort**					
Resolving	402	49 (12%)	1.00 (reference)	1.00 (reference)	1.00 (reference)
Persistent	1358	296 (22%)	1.79 (1.35, 2.37)	1.63 (1.19, 2.24)	1.53 (1.16, 2.02)

Mortality is in-hospital mortality 28 days after enrollment.
Model A: adjusted for age, sex, chronic respiratory disease, and PaO2 to FIO2 ratio on enrollment.
Model B: adjusted for age, sex, chronic respiratory disease, and modified acute physiology and chronic health evaluation on enrollment (APACHE-III score in discovery cohort and APACHE-II score in validation cohort).

Table S4: Relative risk of mortality associated with persistent hypoxemic respiratory failure, redefined at day 4

	Total	Deaths	Relative risk (95% Confidence Interval)		
	N	*N (%)*	*Unadjusted*	*Model A*	*Model B*
**Discovery Cohort**					
Resolving	429	29 (7%)	1.00 (reference)	1.00 (reference)	1.00 (reference)
Persistent	328	68 (21%)	3.07 (2.03, 4.62)	2.86 (1.87, 4.39)	2.21 (1.42, 3.45)
**Validation Cohort**					
Resolving	830	82 (10%)	1.00 (reference)	1.00 (reference)	1.00 (reference)
Persistent	856	189 (22%)	2.23 (1.76, 2.84)	2.32 (1.78, 3.03)	2.10 (1.66, 2.66)

Mortality is in-hospital mortality 28 days after enrollment.
Model A: adjusted for age, sex, chronic respiratory disease, and PaO2 to FIO2 ratio on enrollment.
Model B: adjusted for age, sex, chronic respiratory disease, and modified acute physiology and chronic health evaluation on enrollment (APACHE-III score in discovery cohort and APACHE-II score in validation cohort).

Table S5: Relative risk of mortality associated with persistent hypoxemic respiratory failure among patients with PaO2:FIO2 < 150 on enrollment

	Total	Deaths	Relative risk (95% Confidence Interval)		
	N	*N (%)*	*Unadjusted*	*Model A*	*Model B*
**Discovery Cohort**					
Resolving	110	8 (7%)	1.00 (reference)	1.00 (reference)	1.00 (reference)
Persistent	218	47 (22%)	2.96 (1.45, 6.06)	2.94 (1.45, 5.97)	2.30 (1.11, 4.77)
**Validation Cohort**					
Resolving	170	19 (11%)	1.00 (reference)	1.00 (reference)	1.00 (reference)
Persistent	460	104 (23%)	2.02 (1.28, 3.19)	2.23 (1.43, 3.48)	2.02 (1.30, 3.14)

Mortality is in-hospital mortality 28 days after enrollment.
Model A: adjusted for age, sex, chronic respiratory disease, and PaO2 to FIO2 ratio on enrollment.
Model B: adjusted for age, sex, chronic respiratory disease, and modified acute physiology and chronic health evaluation on enrollment (APACHE-III score in discovery cohort and APACHE-II score in validation cohort).

Table S6: Relative risk of mortality associated with persistent hypoxemic respiratory failure, excluding patients with chronic lung disease

	Total	Deaths	Relative risk (95% Confidence Interval)		
	N	*N (%)*	*Unadjusted*	*Model A*	*Model B*
**Discovery Cohort**					
Resolving	322	26 (8%)	1.00 (reference)	1.00 (reference)	1.00 (reference)
Persistent	298	60 (20%)	2.49 (1.62, 3.84)	2.47 (1.58, 3.87)	1.69 (1.06, 2.68)
**Validation Cohort**					
Resolving	549	56 (10%)	1.00 (reference)	1.00 (reference)	1.00 (reference)
Persistent	910	194 (21%)	2.09 (1.58, 2.76)	2.02 (1.48, 2.76)	1.83 (1.39, 2.40)

Mortality is in-hospital mortality 28 days after enrollment.
Model A: adjusted for age, sex, and PaO2 to FIO2 ratio on enrollment.
Model B: adjusted for age, sex, and modified acute physiology and chronic health evaluation on enrollment (APACHE-III score in discovery cohort and APACHE-II score in validation cohort).

SUPPLEMENTAL TABLES – DIFFERENCES IN CIRCULATING BIOMARKERS

Table S7: Median biomarker concentrations by persistent and resolving hypoxemic respiratory failure

	Discovery Cohort			Validation Cohort		
**Biomarker**	**Resolving**	**Persistent**	***P* value**	**Resolving**	**Persistent**	***P* value**
IL-6	96 (37-237)	242 (79-683)	<0.001	38 (15-127)	80 (29-350)	<0.001
IL-8	11 (5-26)	18 (9-38)	<0.001	16 (9-38)	23 (11-81)	<0.001
sTNFR-1	7694 (4826-14129)	11443 (6744-18735)	<0.001	2874 (1733-5139)	2638 (1587-5354)	0.44
sFas	11394 (8063-15790)	12988 (9717-18202)	<0.001	n.a.		
IL17A	3 (1-8)	4 (2-14)	0.001	n.a.		
G-CSF	26 (13-49)	38 (18-135)	<0.001	n.a.		
Ang-2	13246 (6595-24508)	24112 (11010-54941)	<0.001	4186 (2731-7806)	6047 (3863-10635)	<0.001
Ang-1	4948 (1951-9541)	3615 (1523-7026)	<0.001	n.a.		
sVCAM-1	533 (392-802)	572 (445-845)	0.034	n.a.		

Abbreviations: IL-6 = interleukin-6; IL-8 = interleukin-8; sTNFR-1 = soluble tumor necrosis factor receptor-1; sFas = soluble Fas; IL-17A = interleukin-17A; G-CSF = granulocyte-colony stimulating factor; Ang-2 = angiopoietin-2; Ang-1 = angiopoietin-1; sVCAM-1 = soluble vascular cell adhesion protein-1; n.a. = not measured in validation cohort. Biomarker concentrations were measured in plasma obtained on enrollment, and expressed here as median (interquartile range). All concentrations are in pg/mL, except sVCAM-1 which is in ng/mL. *P* values for Mann Whitney U tests.

Table S8. Fold-differences in biomarker concentrations between persistent and resolving hypoxemic respiratory failure

	Fold-difference (95% Confidence Interval)		
	Unadjusted	*Model A*	*Model B*
**Discovery Cohort**			
IL-6	2.65 (2.02, 3.48)*	2.74 (2.04, 3.67)*	2.44 (1.84, 3.23)*
IL-8	1.68 (1.34, 2.11)*	1.76 (1.39, 2.24)*	1.64 (1.30, 2.07)*
sTNFR-1	1.39 (1.20, 1.61)*	1.48 (1.25, 1.74)*	1.25 (1.08, 1.46)*
sFas	1.17 (1.08, 1.28)*	1.20 (1.10, 1.31)*	1.11 (1.02, 1.20)*
IL17A	1.50 (1.17, 1.92)*	1.55 (1.20, 2.01)*	1.28 (0.98, 1.66)
G-CSF	1.95 (1.52, 2.49)*	2.05 (1.59, 2.65)*	1.85 (1.43, 2.40)*
Ang-2	1.77 (1.52, 2.08)*	1.78 (1.51, 2.11)*	1.49 (1.27, 1.74)*
Ang-1	0.72 (0.59, 0.88)*	0.76 (0.62, 0.94)*	0.82 (0.67, 1.01)
sVCAM-1	1.11 (1.01, 1.21)*	1.12 (1.02, 1.24)*	1.06 (0.96, 1.16)*
**Validation Cohort**			
IL-6	2.36 (1.81, 3.09)*	2.04 (1.48, 2.81)*	2.04 (1.57, 2.66)*
IL-8	1.74 (1.44, 2.10)*	1.60 (1.29, 1.99)*	1.49 (1.24, 1.79)*
sTNFR-1	0.96 (0.84, 1.10)	0.99 (0.84, 1.15)	0.86 (0.76, 0.97)*
Ang-2	1.42 (1.22, 1.66)*	1.37 (1.17, 1.61)*	1.32 (1.14, 1.53)*

Abbreviations: IL-6 = interleukin-6; IL-8 = interleukin-8; sTNFR-1 = soluble tumor necrosis factor receptor-1; sFas = soluble Fas; IL-17A = interleukin-17A; G-CSF = granulocyte-colony stimulating factor; Ang-2 = angiopoietin-2; Ang-1 = angiopoietin-1; sVCAM-1 = soluble vascular cell adhesion protein-1.
Fold-differences reflect ratio of geometric mean concentrations among patients with persistent hypoxemic respiratory failure, to patients with resolving hypoxemic respiratory failure.
Model A: adjusted for age, sex, chronic respiratory disease, and PaO2 to FIO2 ratio on enrollment.
Model B: adjusted for age, sex, chronic respiratory disease, and modified acute physiology and chronic health evaluation on enrollment (APACHE-III score in discovery cohort and APACHE-II score in validation cohort).

SUPPLEMENTAL TABLES – SENSITIVITY ANALYSES OF CIRCULATING BIOMARKERS

Table S9: Median and fold-difference in biomarker values between persistent and resolving hypoxemic respiratory failure, inclusive of patients who died before day 3

		**Resolving**	**Persistent**		
	N	*Median (IQR)*	*Median (IQR)*	*P value*	*Fold-difference*
**Discovery Cohort**					
IL-6	550	96 (37-237)	248 (84-751)	<0.001	2.55 (1.93, 3.38)
IL-8	550	11 (5-26)	18 (9-42)	<0.001	1.78 (1.40, 2.26)
sTNFR-1	550	7694 (4826-14129)	11671 (6784-19299)	<0.001	1.29 (1.11, 1.51)
sFas	584	11394 (8063-15790)	13127 (9673-18373)	<0.001	1.11 (1.02, 1.21)
IL17A	454	3 (1-8)	4 (2-14)	0.001	1.29 (0.99, 1.67)
G-CSF	550	26 (13-49)	38 (19-146)	<0.001	1.97 (1.52, 2.56)
Ang-2	584	13246 (6595-24508)	24412 (11260-56338)	<0.001	1.53 (1.31, 1.80)
Ang-1	579	4948 (1951-9541)	3376 (1523-7026)	<0.001	0.81 (0.66, 0.99)
sVCAM-1	584	533 (392-802)	572 (446-849)	0.025	1.06 (0.96, 1.17)
**Validation Cohort**					
IL-6	848	38 (15-127)	86 (29-430)	<0.001	2.29 (1.75, 2.98)
IL-8	1011	16 (9-38)	25 (12-99)	<0.001	1.61 (1.33, 1.93)
sTNFR-1	806	2874 (1733-5139)	2901 (1640-5845)	0.85	0.89 (0.79, 1.01)
Ang-2	484	4208 (2731-7806)	6250 (3976-11205)	<0.001	1.34 (1.16, 1.55)

Abbreviations: IL-6 = interleukin-6; IL-8 = interleukin-8; sTNFR-1 = soluble tumor necrosis factor receptor-1; sFas = soluble Fas; IL-17A = interleukin-17A; G-CSF = granulocyte-colony stimulating factor; Ang-2 = angiopoietin-2; Ang-1 = angiopoietin-1; sVCAM-1 = soluble vascular cell adhesion protein-1.
N specifies number of patients who had that biomarker measured. Biomarker concentrations were measured in plasma obtained on enrollment, and expressed here as median (interquartile range). All concentrations are in pg/mL, except sVCAM-1 which is in ng/mL.
P value is for Mann Whitney U tests.
Fold difference is adjusted for age, sex, chronic respiratory disease and modified acute physiology and chronic health evaluation on enrollment score (APACHE-III score in discovery cohort and APACHE-II score in validation cohort), and reflects ratio of geometric mean concentration among patients with persistent hypoxemic respiratory failure, to patients with resolving hypoxemic respiratory failure.

Table S10: Median and fold-difference in biomarker values between persistent and resolving hypoxemic respiratory failure, redefined at day 2

		Resolving	Persistent		
	N	*Median (IQR)*	*Median (IQR)*	*P value*	*Fold-difference*
**Discovery Cohort**					
IL-6	544	90 (36-205)	218 (79-632)	<0.001	2.31 (1.74, 3.07)
IL-8	544	11 (5-29)	17 (9-38)	<0.001	1.55 (1.23, 1.95)
sTNFR-1	544	7653 (4709-13475)	11527 (6702-18514)	<0.001	1.23 (1.06, 1.44)
sFas	578	11245 (8102-15550)	13133 (9693-18115)	<0.001	1.10 (1.00, 1.20)
IL17A	449	3 (1-7)	5 (2-15)	<0.001	1.45 (1.13, 1.86)
G-CSF	544	26 (14-52)	34 (17-115)	<0.001	1.53 (1.18, 1.98)
Ang-2	578	12449 (6506-24178)	23141 (10650-54512)	<0.001	1.43 (1.22, 1.68)
Ang-1	573	5475 (2072-10091)	3370 (1470-6778)	<0.001	0.77 (0.63, 0.94)
sVCAM-1	578	529 (393-766)	576 (443-864)	0.010	1.07 (0.97, 1.18)
**Validation Cohort**					
IL-6	833	41 (15-107)	75 (26-333)	<0.001	1.82 (1.33, 2.49)
IL-8	993	16 (10-43)	21 (11-70)	0.034	1.12 (0.88, 1.42)
sTNFR-1	793	2737 (1720-4939)	2857 (1634-5535)	0.93	0.90 (0.78, 1.05)
Ang-2	473	4797 (3147-8373)	5666 (3599-10445)	0.13	1.02 (0.85, 1.21)

Abbreviations: IL-6 = interleukin-6; IL-8 = interleukin-8; sTNFR-1 = soluble tumor necrosis factor receptor-1; sFas = soluble Fas; IL-17A = interleukin-17A; G-CSF = granulocyte-colony stimulating factor; Ang-2 = angiopoietin-2; Ang-1 = angiopoietin-1; sVCAM-1 = soluble vascular cell adhesion protein-1.
N specifies number of patients who had that biomarker measured. Biomarker concentrations were measured in plasma obtained on enrollment, and expressed here as median (interquartile range). All concentrations are in pg/mL, except sVCAM-1 which is in ng/mL.
P value is for Mann Whitney U tests.
Fold difference is adjusted for age, sex, chronic respiratory disease and modified acute physiology and chronic health evaluation on enrollment score (APACHE-III score in discovery cohort and APACHE-II score in validation cohort), and reflects ratio of geometric mean concentration among patients with persistent hypoxemic respiratory failure, to patients with resolving hypoxemic respiratory failure.

Table S11: Median and fold-difference in biomarker values between persistent and resolving hypoxemic respiratory failure, redefined at day 4

		Resolving	Persistent		
	N	*Median (IQR)*	*Median (IQR)*	*P value*	*Fold-difference*
**Discovery Cohort**					
IL-6	529	100 (43-246)	242 (78-632)	<0.001	2.15 (1.61, 2.87)
IL-8	529	11 (5-25)	18 (9-37)	<0.001	1.58 (1.26, 2.00)
sTNFR-1	529	7694 (4828-14382)	11386 (6784-18893)	<0.001	1.25 (1.08, 1.45)
sFas	563	11337 (8093-15683)	13271 (9848-18587)	<0.001	1.14 (1.04, 1.24)
IL17A	440	3 (1-8)	5 (2-16)	<0.001	1.37 (1.04, 1.81)
G-CSF	529	26 (14-52)	37 (18-136)	<0.001	1.79 (1.36, 2.37)
Ang-2	563	13463 (6776-25781)	23992 (10762-55332)	<0.001	1.41 (1.20, 1.67)
Ang-1	558	4900 (1975-9545)	3646 (1552-7008)	<0.001	0.81 (0.65, 1.00)
sVCAM-1	563	526 (388-779)	619 (461-888)	0.002	1.10 (0.99, 1.22)
**Validation Cohort**					
IL-6	786	43 (17-133)	93 (30-404)	<0.001	2.12 (1.64, 2.74)
IL-8	942	16 (9-38)	23 (11-87)	<0.001	1.58 (1.32, 1.90)
sTNFR-1	749	2787 (1683-5187)	2590 (1570-5314)	0.49	0.88 (0.78, 0.99)
Ang-2	451	4426 (2946-7806)	6191 (3809-11012)	<0.001	1.31 (1.13, 1.51)

Abbreviations: IL-6 = interleukin-6; IL-8 = interleukin-8; sTNFR-1 = soluble tumor necrosis factor receptor-1; sFas = soluble Fas; IL-17A = interleukin-17A; G-CSF = granulocyte-colony stimulating factor; Ang-2 = angiopoietin-2; Ang-1 = angiopoietin-1; sVCAM-1 = soluble vascular cell adhesion protein-1.
N specifies number of patients who had that biomarker measured. Biomarker concentrations were measured in plasma obtained on enrollment, and expressed here as median (interquartile range). All concentrations are in pg/mL, except sVCAM-1 which is in ng/mL.
P value is for Mann Whitney U tests.
Fold difference is adjusted for age, sex, chronic respiratory disease and modified acute physiology and chronic health evaluation on enrollment score (APACHE-III score in discovery cohort and APACHE-II score in validation cohort), and reflects ratio of geometric mean concentration among patients with persistent hypoxemic respiratory failure, to patients with resolving hypoxemic respiratory failure.

Table S12: Median and fold-difference in biomarker values between persistent and resolving hypoxemic respiratory failure, among patients with PaO2:FIO2 < 150 on enrollment

		**Resolving**	**Persistent**		
	N	*Median (IQR)*	*Median (IQR)*	*P value*	*Fold-difference*
**Discovery Cohort**					
IL-6	229	77 (20-207)	271 (86-708)	<0.001	3.85 (2.43, 6.09)
IL-8	229	7 (4-13)	19 (9-39)	<0.001	2.73 (1.89, 3.96)
sTNFR-1	229	6469 (4259-12063)	11223 (6784-18893)	<0.001	1.55 (1.18, 2.03)
sFas	242	10298 (7370-15170)	13138 (9595-18115)	<0.001	1.23 (1.08, 1.40)
IL17A	181	2 (1-7)	5 (2-18)	<0.001	1.75 (1.19, 2.58)
G-CSF	229	19 (12-39)	32 (18-141)	<0.001	2.63 (1.81, 3.84)
Ang-2	242	10886 (5202-18464)	27181 (12604-56742)	<0.001	2.05 (1.60, 2.61)
Ang-1	239	5735 (2202-10425)	3141 (1190-6295)	<0.001	0.63 (0.46, 0.88)
sVCAM-1	242	484 (344-744)	560 (448-829)	0.011	1.15 (0.98, 1.36)
**Validation Cohort**					
IL-6	357	40 (13-118)	78 (28-327)	<0.001	2.27 (1.38, 3.72)
IL-8	397	14 (7-34)	19 (10-77)	0.001	1.71 (1.23, 2.40)
sTNFR-1	341	2387 (1773-5187)	2272 (1541-4378)	0.23	0.83 (0.68, 1.00)
Ang-2	176	4306 (2701-7524)	7155 (4197-11587)	0.002	1.53 (1.18, 1.99)

Abbreviations: IL-6 = interleukin-6; IL-8 = interleukin-8; sTNFR-1 = soluble tumor necrosis factor receptor-1; sFas = soluble Fas; IL-17A = interleukin-17A; G-CSF = granulocyte-colony stimulating factor; Ang-2 = angiopoietin-2; Ang-1 = angiopoietin-1; sVCAM-1 = soluble vascular cell adhesion protein-1.

N specifies number of patients who had that biomarker measured. Biomarker concentrations were measured in plasma obtained on enrollment, and expressed here as median (interquartile range). All concentrations are in pg/mL, except sVCAM-1 which is in ng/mL.

P value is for Mann Whitney U tests.

Fold difference is adjusted for age, sex, chronic respiratory disease and modified acute physiology and chronic health evaluation on enrollment score (APACHE-III score in discovery cohort and APACHE-II score in validation cohort), and reflects ratio of geometric mean concentration among patients with persistent hypoxemic respiratory failure, to patients with resolving hypoxemic respiratory failure.

Table S13: Median and fold-difference in biomarker values between persistent and resolving hypoxemic respiratory failure, excluding patients with chronic lung disease

		Resolving	Persistent		
	N	*Median (IQR)*	*Median (IQR)*	*P value*	*Fold-difference*
**Discovery Cohort**					
IL-6	436	99 (43-237)	254 (102-648)	<0.001	2.61 (1.93, 3.52)
IL-8	436	11 (5-26)	19 (11-41)	<0.001	1.83 (1.41, 2.37)
sTNFR-1	436	7802 (4808-14129)	11857 (7111-19654)	<0.001	1.39 (1.18, 1.63)
sFas	466	11394 (8063-15790)	13485 (9693-18957)	<0.001	1.14 (1.04, 1.25)
IL17A	367	3 (1-8)	5 (2-15)	<0.001	1.32 (0.99, 1.75)
G-CSF	436	26 (14-49)	38 (20-141)	<0.001	1.97 (1.48, 2.62)
Ang-2	466	13237 (6733-24320)	24159 (11622-55332)	<0.001	1.55 (1.30, 1.84)
Ang-1	463	4863 (1832-9549)	3209 (1470-6522)	0.001	0.80 (0.64, 1.01)
sVCAM-1	466	532 (388-783)	601 (456-888)	0.003	1.13 (1.01, 1.25)
**Validation Cohort**					
IL-6	678	43 (17-134)	81 (29-346)	<0.001	1.87 (1.40, 2.50)
IL-8	822	16 (9-38)	23 (11-87)	<0.001	1.53 (1.24, 1.88)
sTNFR-1	643	2704 (1720-5034)	2684 (1578-5135)	0.54	0.86 (0.75, 0.99)
Ang-2	401	4088 (2644-7389)	6024 (3854-10554)	<0.001	1.34 (1.15, 1.57)

Abbreviations: IL-6 = interleukin-6; IL-8 = interleukin-8; sTNFR-1 = soluble tumor necrosis factor receptor-1; sFas = soluble Fas; IL-17A = interleukin-17A; G-CSF = granulocyte-colony stimulating factor; Ang-2 = angiopoietin-2; Ang-1 = angiopoietin-1; sVCAM-1 = soluble vascular cell adhesion protein-1.
N specifies number of patients who had that biomarker measured. Biomarker concentrations were measured in plasma obtained on enrollment, and expressed here as median (interquartile range). All concentrations are in pg/mL, except sVCAM-1 which is in ng/mL.
P value is for Mann Whitney U tests.
Fold difference is adjusted for age, sex, and modified acute physiology and chronic health evaluation on enrollment score (APACHE-III score in discovery cohort and APACHE-II score in validation cohort), and reflects ratio of geometric mean concentration among patients with persistent hypoxemic respiratory failure, to patients with resolving hypoxemic respiratory failure.

SUPPLEMENTAL TABLES – RELATIONSHIPS BETWEEN PERSISTENT/RESOLVING HRF AND ARDS

Table S14: Cohort descriptions stratified by persistent/resolving hypoxemic respiratory failure and +/- acute respiratory distress syndrome (ARDS)

	Discovery Cohort					Validation Cohort				
	Resolving		Persistent			Resolving		Persistent		
	-ARDS N=296	*+ARDS* N=86	*-ARDS* N=186	*+ARDS* N=200	*P* value	*-ARDS* N=515	*+ARDS* N=168	*-ARDS* N=559	*+ARDS* N=473	*P* value
**Demographics**										
Age, years	53 (45-63)	56 (45-65)	53 (43-62)	54 (46-67)	0.55	56 (43-66)	53 (37-65)	54 (43-65)	51 (39-63)	0.015
Female	114 (39%)	30 (35%)	73 (39%)	56 (28%)	0.065	198 (38%)	66 (39%)	165 (30%)	192 (41%)	<0.001
**Baseline Comorbidities**										
Diabetes	87 (29%)	26 (30%)	58 (31%)	54 (27%)	0.83	136 (26%)	50 (30%)	147 (26%)	111 (23%)	0.41
Cirrhosis	33 (11%)	11 (13%)	18 (10%)	26 (13%)	0.75	50 (10%)	11 (7%)	41 (7%)	33 (7%)	0.31
Chronic respiratory disease	49 (17%)	11 (13%)	46 (25%)	42 (21%)	0.053	107 (21%)	27 (16%)	68 (12%)	54 (11%)	<0.001
Heart Failure	28 (9%)	13 (15%)	19 (10%)	24 (12%)	0.47	57 (11%)	17 (10%)	54 (10%)	29 (6%)	0.050
**ICU events on enrollment**										
Type of ICU					0.058					<0.001
Medical	197 (67%)	63 (73%)	121 (65%)	152 (76%)		217 (42%)	88 (53%)	185 (33%)	224 (47%)	
Surgical	99 (33%)	23 (27%)	65 (35%)	48 (24%)		138 (27%)	26 (16%)	111 (20%)	81 (17%)	
Trauma						157 (31%)	53 (32%)	261 (47%)	168 (36%)	
Shock	53 (18%)	34 (40%)	79 (42%)	112 (56%)	<0.001	195 (38%)	66 (39%)	274 (49%)	258 (55%)	<0.001
Sepsis	225 (76%)	73 (85%)	151 (81%)	179 (90%)	0.002	169 (33%)	92 (55%)	201 (36%)	274 (58%)	<0.001
Pneumonia	51 (17%)	30 (35%)	49 (26%)	94 (47%)	<0.001	114 (22%)	84 (50%)	147 (26%)	238 (50%)	<0.001
**Illness severity on enrollment**										
PaO2:FIO2	192 (143-260)	183 (136-246)	167 (112-228)	105 (69-165)	<0.001	192 (138-243)	171 (129-218)	162 (114-220)	122 (80-183)	<0.001
SOFA	4 (2-6)	4 (3-6)	5 (3-7)	6 (4-7)	<0.001	8 (7-10)	9 (8-11)	9 (8-11)	10 (8-12)	<0.001
APACHE-III	49 (35-69)	68 (49-84)	62 (45-86)	74 (55-92)	<0.001	n.a.				
APACHE-II	n.a.					25 (20-31)	27 (23-33)	28 (22-33)	29 (25-34)	<0.001
**Clinical Outcomes**										
VFD	26 (24-27)	25 (13-26)	17 (1-22)	11 (0-20)	<0.001	26 (24-26)	25 (11-26)	18 (1-23)	15 (0-21)	<0.001
LOS, days	9 (6-18)	11 (7-26)	17 (11-32)	21 (14-34)	<0.001	10 (7-17)	11 (7-18)	16 (10-26)	16 (11-27)	<0.001
Mortality	22 (7%)	9 (10%)	28 (15%)	49 (25%)	<0.001	37 (7%)	33 (20%)	114 (20%)	116 (25%)	<0.001

Abbreviations: PaO2:FIO2 = PaO2 to FIO2 ratio; SOFA = sequential organ failure assessment; APACHE = acute physiology and chronic health evaluation; ICU = intensive care unit; VFD = ventilator free days; LOS = hospital length of stay.

Continuous variables are expressed as median (interquartile range), and compared using Kruskal-Wallis tests. Categorical variables are expressed as number (percentage), and were compared using Chi square tests.

Table S15: Biomarker concentrations by persistent and resolving hypoxemic respiratory failure, stratified by acute respiratory distress syndrome (+/-ARDS)

	N	Resolving		Persistent		*P*
		-ARDS	*+ARDS*	*-ARDS*	*+ARDS*	value
**Discovery Cohort**						
IL-6	538	94 (33-231)	102 (49-241)	192 (63-619)	288 (100-708)	<0.001
IL-8	538	10 (5-25)	11 (7-30)	17 (10-42)	18 (9-29)	<0.001
sTNFR-1	538	7731 (4697-13641)	7590 (5477-15724)	11158 (6763-20599)	11622 (6744-18359)	<0.001
sFas	572	11077 (7760-15296)	12787 (8903-16676)	13184 (10119-18847)	12161 (8897-16904)	<0.001
IL17A	443	3 (1-8)	3 (1-6)	4 (2-15)	4 (2-14)	0.014
G-CSF	538	26 (13-49)	25 (15-55)	38 (19-128)	36 (18-136)	<0.001
Ang-2	572	13247 (6422-24375)	13245 (6843-26050)	22263 (8504-47239)	27089 (13667-63816)	<0.001
Ang-1	567	4919 (1830-10091)	4992 (2819-7675)	3212 (1828-7883)	3718 (1244-6286)	0.004
sVCAM-1	572	544 (392-789)	515 (382-908)	564 (448-888)	575 (435-811)	0.18
**Validation Cohort**						
IL-6	804	38 (15-122)	38 (17-127)	109 (35-530)	75 (26-309)	<0.001
IL-8	961	16 (12-41)	14 (7-31)	28 (16-81)	21 (9-81)	<0.001
sTNFR-1	767	3228 (2054-5866)	2358 (1517-4470)	4499 (2370-7702)	2243 (1372-4260)	<0.001
Ang-2	458	4055 (2556-7010)	4533 (2874-10397)	5523 (3962-8499)	7053 (3826-12322)	<0.001

Abbreviations: IL-6 = interleukin-6; IL-8 = interleukin-8; sTNFR-1 = soluble tumor necrosis factor receptor-1; sFas = soluble Fas; IL-17A = interleukin-17A; G-CSF = granulocyte-colony stimulating factor; Ang-2 = angiopoietin-2; Ang-1 = angiopoietin-1; sVCAM-1 = soluble vascular cell adhesion protein-1.
Biomarker concentrations were measured in plasma obtained on enrollment, and expressed here as median (interquartile range). All concentrations are in pg/mL, except sVCAM-1 which is in ng/mL.
P values for Kruskal Wallis tests.
N refers to number of patients who had the specified biomarker measured in each cohort.

Table S16: Pairwise comparisons between 4 strata of persistent/resolving hypoxemic respiratory failure (HRF) and acute respiratory distress syndrome (+/-ARDS)

	**Fold-difference (95% Confidence Interval) between groups**					
	Persistent HRF/-ARDS vs. Resolving HRF/-ARDS*	Resolving HRF/+ARDS vs. Resolving HRF/-ARDS*	Persistent HRF/+ARDS vs. Resolving HRF/-ARDS*	Resolving HRF/+ARDS vs. Persistent HRF/-ARDS	Persistent HRF/+ARDS vs. Persistent HRF/-ARDS	Persistent HRF/+ARDS vs. Resolving HRF/+ARDS
**Discovery Cohort**						
IL-6	**2.42 (1.49, 3.91)**	1.22 (0.69, 2.17)	**2.75 (1.71, 4.41)**	**0.51 (0.27, 0.95)**	1.14 (0.68, 1.91)	**2.25 (1.20, 4.20)**
IL-8	**1.84 (1.22, 2.77)**	1.12 (0.69, 1.82)	**1.52 (1.03, 2.25)**	0.61 (0.36, 1.02)	0.83 (0.54, 1.26)	1.36 (0.82, 2.26)
sTNFR-1	**1.32 (1.02, 1.71)**	1.01 (0.76, 1.35)	1.18 (0.90, 1.55)	0.77 (0.58, 1.02)	0.89 (0.70, 1.14)	1.17 (0.87, 1.56)
sFas	**1.19 (1.04, 1.37)**	1.04 (0.87, 1.25)	1.03 (0.88, 1.20)	0.87 (0.73, 1.05)	0.86 (0.74, 1.00)	0.98 (0.81, 1.19)
IL17A	1.26 (0.81, 1.95)	0.78 (0.46, 1.32)	1.14 (0.73, 1.79)	0.62 (0.35, 1.10)	0.91 (0.56, 1.45)	1.46 (0.82, 2.62)
G-CSF	**2.03 (1.31, 3.15)**	1.23 (0.74, 2.06)	**1.86 (1.18, 2.93)**	0.61 (0.33, 1.11)	0.91 (0.53, 1.57)	1.51 (0.81, 2.80)
Ang-2	1.31 (0.99, 1.73)	0.90 (0.65, 1.24)	**1.64 (1.25, 2.15)**	**0.68 (0.48, 0.97)**	1.25 (0.94, 1.66)	**1.83 (1.31, 2.56)**
Ang-1	0.92 (0.65, 1.30)	1.37 (0.95, 1.98)	0.85 (0.58, 1.25)	**1.49 (1.01, 2.19)**	0.93 (0.63, 1.35)	**0.62 (0.42, 0.93)**
sVCAM-1	1.08 (0.92, 1.27)	0.96 (0.77, 1.18)	1.00 (0.84, 1.19)	0.88 (0.71, 1.10)	0.92 (0.77, 1.10)	1.05 (0.83, 1.31)
**Validation Cohort**						
IL-6	**3.01 (1.67, 5.42)**	1.12 (0.64, 1.94)	**1.89 (1.18, 3.03)**	**0.37 (0.21, 0.66)**	0.63 (0.38, 1.03)	**1.69 (1.07, 2.68)**
IL-8	**1.68 (1.20, 2.36)**	0.75 (0.51, 1.11)	1.16 (0.85, 1.58)	**0.45 (0.30, 0.68)**	**0.69 (0.49, 0.97)**	**1.54 (1.05, 2.26)**
sTNFR-1	1.13 (0.89, 1.44)	0.79 (0.61, 1.02)	0.66 (0.54, 0.81)	**0.69 (0.53, 0.91)**	**0.58 (0.47, 0.71)**	0.84 (0.67, 1.05)
Ang-2	**1.33 (1.04, 1.70)**	1.21 (0.86, 1.69)	**1.47 (1.13, 1.90)**	0.91 (0.65, 1.26)	1.10 (0.86, 1.41)	1.22 (0.87, 1.70)

Abbreviations: IL-6 = interleukin-6; IL-8 = interleukin-8; sTNFR-1 = soluble tumor necrosis factor receptor-1; sFas = soluble Fas; IL-17A = interleukin-17A; G-CSF = granulocyte-colony stimulating factor; Ang-2 = angiopoietin-2; Ang-1 = angiopoietin-1; sVCAM-1 = soluble vascular cell adhesion protein-1.
This graph reflects fold-differences in biomarker concentrations between the groups listed at the top.
The fold differences are adjusted for age, sex, chronic respiratory disease and APACHE score (APACHE-III in the discovery cohort; APACHE-II in the validation cohort).
*These comparisons are the same comparisons presented in the main paper, with the exception that these confidence intervals have Bonferroni correction.
Bolded estimates are significant at Bonferroni corrected $P < 0.05$.

Table S17: Hypoinflammatory and hyperinflammatory subphenotypes among patients with acute hypoxemic respiratory failure

	Discovery			Validation		
	Hypoinflammatory	Hyperinflammatory	*P* value	Hypoinflammatory	Hyperinflammatory	*P* value
	N=407	N=131		N=613	N=151	
Age	54 (45-65)	53 (44-62)	0.27	54 (40-65)	56 (44-66)	0.25
Female	147 (36%)	37 (28%)	0.098	228 (37%)	66 (44%)	0.14
Vasopressors	124 (30%)	53 (40%)	0.034	279 (46%)	108 (72%)	<0.001
ARDS	86 (21%)	30 (23%)	0.67	351 (57%)	80 (53%)	0.34
PaO2:FIO2	165 (107-233)	170 (104-248)	0.64	139 (88-196)	153 (91-198)	0.33
HCO3	22 (19-25)	15 (11-19)	<0.001	22 (19-24)	17 (13-21)	<0.001
IL-8	11 (5-18)	55 (26-136)	<0.001	14 (7-29)	218 (81-947)	<0.001
sTNFR-1	7317 (4900-12229)	20209 (13173-36071)	<0.001	2261 (1477-3856)	7991 (4470-14606)	<0.001
IL-6	104 (43-252)	444 (158-1587)	<0.001	45 (19-118)	490 (130-2563)	<0.001
Ang-2	13430 (6872-25781)	48055 (22834-77419)	<0.001	5016 (3188-9109)	8909 (5800-17253)	<0.001
VFD	23 (10-26)	13 (0-23)	<0.001	20 (6-25)	8 (0-22)	<0.001
Mortality	38 (9%)	39 (30%)	<0.001	117 (19%)	52 (34%)	<0.001

Abbreviations: PaO2:FIO2 = PaO2 to FIO2 ratio; HCO3 = serum bicarbonate in mmol/L; IL-8 = interleukin-8 in pg/mL; sTNFR-1 = soluble tumor necrosis factor receptor-1 in pg/mL; IL-6 = interleukin-6 in pg/mL; Ang-2 = angiopoietin-2; VFD = ventilator free days.
Continuous variables are expressed as median (interquartile range), and compared using Mann Whitney U tests. Categorical variables are expressed as number (percentage), and were compared using Chi square tests.
